# Bile acids as inflammatory mediators and modulators of intestinal permeability

**DOI:** 10.3389/fimmu.2022.1021924

**Published:** 2022-12-07

**Authors:** Nathan Calzadilla, Shane M. Comiskey, Pradeep K. Dudeja, Seema Saksena, Ravinder K. Gill, Waddah A. Alrefai

**Affiliations:** ^1^ Division of Gastroenterology and Hepatology, Department of Medicine, University of Illinois, Chicago, IL, United States; ^2^ Department of Bioengineering, University of Illinois, Chicago, IL, United States; ^3^ Research and Development, Jesse Brown VA Medical Center, Chicago, IL, United States

**Keywords:** bile acids, intestinal epithelial tight junction proteins, Crohn’s disease, ulcerative, bile acid receptors

## Abstract

Bile acids are critical for the digestion and absorption of lipids and fat-soluble vitamins; however, evidence continues to emerge supporting additional roles for bile acids as signaling molecules. After they are synthesized from cholesterol in the liver, primary bile acids are modified into secondary bile acids by gut flora contributing to a diverse pool and making the composition of bile acids highly sensitive to alterations in gut microbiota. Disturbances in bile acid homeostasis have been observed in patients with Inflammatory Bowel Diseases (IBD). In fact, a decrease in secondary bile acids was shown to occur because of IBD-associated dysbiosis. Further, the increase in luminal bile acids due to malabsorption in Crohn’s ileitis and ileal resection has been implicated in the induction of diarrhea and the exacerbation of inflammation. A causal link between bile acid signaling and intestinal inflammation has been recently suggested. With respect to potential mechanisms related to bile acids and IBD, several studies have provided strong evidence for direct effects of bile acids on intestinal permeability in porcine and rodent models as well as in humans. Interestingly, different bile acids were shown to exert distinct effects on the inflammatory response and intestinal permeability that require careful consideration. Such findings revealed a potential effect for changes in the relative abundance of different bile acids on the induction of inflammation by bile acids and the development of IBD. This review summarizes current knowledge about the roles for bile acids as inflammatory mediators and modulators of intestinal permeability mainly in the context of inflammatory bowel diseases.

## 1 Introduction

Bile acids are synthesized in the liver from cholesterol and secreted into the intestine where they serve to emulsify and facilitate the absorption of dietary lipids. Approximately 5% of the bile acid pool is eliminated in the stool and serves as a major pathway for eliminating cholesterol from the body (1). Most bile acids are reabsorbed in the distal ileum and are transported back to the liver for re-secretion, establishing their enterohepatic circulation. In addition to being lipid emulsifiers in the intestinal lumen, bile acids also activate specific receptors with varying physiological effects in several organ systems throughout the body (2). Different receptors for bile acids have been identified in various immune cells throughout the gastrointestinal tract. Strong evidence suggests that the activation of bile acid receptors in immune cells modulates the immune responses and alters inflammatory reactions (3). Also, bile acids have been shown to trigger specific signaling pathways that disrupt barrier function and increase intestinal epithelial permeability. The leaky gut condition induced by bile acids could be attributed to the pathophysiology of inflammation in inflammatory bowel diseases (IBD) (4).

The bile acid pool is highly conserved and is composed of different types of bile acids, each having a specific affinity to certain bile acid receptors. Interestingly, secondary bile acids are generated in the intestine and therefore the gut microbiota is crucial for expanding the metabolic profile of the bile acid pool (5). The abundance of certain bile acids and the overall composition of the bile acid pool ultimately determines its functional capacity. Indeed, there is strong evidence to suggest that gut dysbiosis in inflammatory bowel disease (IBD) leads to alterations in the bile acid pool that may affect the severity of the disease and the development of associated pathologies (6). Over the years, this topic has generated extensive interest leading to many seminal studies investigating the roles of bile acid homeostasis and bile acids in the pathophysiology of IBD. In this review, we will highlight recent advances that have enhanced our understanding about the physiological roles of bile acids and how they contribute to IBD by altering the immune responses and epithelial barrier function in the intestine.

## 2 Bile acid homeostasis

### 2.1 Bile acid synthesis

The liver is the primary site for bile acid synthesis in most vertebrates. There are exceptions, however, such as basal jawless vertebrate sea lampreys (*Petromyzon marrinus*) where bile acid synthesis occurs in the liver and intestine (7). The two primary bile acids synthesized in the liver are cholic acid (CA) and chenodeoxycholic acid (CDCA). The synthesis of CA and CDCA involves several cytochrome P450 (CYP) enzymes, with CYP7A1 acting as the rate-limiting enzyme of the process (8). Following their synthesis, bile acids are conjugated to taurine or glycine, a process mediated by the bile acid-coenzyme A ligase (BAL) and the bile acid-coenzyme A:amino acid N-acyltransferase (BAT) (9). Bile acids undergo several transformations by bacteria in the colon, including deconjugation and the formation of secondary bile acids. The removal of amino acids is controlled by a family of bile salt hydrolases (BSHs), while the generation of the secondary bile acids deoxycholic acid (DCA) and lithocholic acid (LCA) is mediated by 7α-dehydroxylation enzymes (10).

The average bile acid pool in healthy humans is 3-5 grams (11). Approximately 95% of the bile acid pool is actively reabsorbed in the distal ileum with approximately 5% of the pool (~ 0.5 g) being excreted into the feces.

The hormone-like protein Fibroblast growth factor 19 (FGF19 in humans, FGF15 in rodents) plays a key role in bile acid homeostasis by serving as a negative regulator of bile acid synthesis. Following active absorption in the ileum, bile acids activate the intestinal farnesoid X receptor (FXR), leading to the induction of FGF19 expression. FGF19 is then secreted into the portal blood stream and eventually suppresses CYP7A1 and bile acid synthesis in the liver (12). Interestingly, studies in primary human hepatocytes have shown that treatment with CDCA following knockdown of FGF19 is sufficient to downregulate CYP7A1 expression, suggesting that bile acids also can regulate their own synthesis without the negative feedback of FGF19 (13).

While bile acids are preserved in the enterohepatic circulation, a small percentage enter the systemic circulation where they modulate the function of different organ systems, including the central nervous system (CNS) (14). Indeed, bile acids can cross the blood-brain barrier (BBB) and are found in both mouse and human brains. In this regard, bile acids have been recently characterized as having both neuroprotective and cytotoxic effects in the brain, with the 12α-hydroxylated bile acids cholic acid (CA) and deoxycholic acid (DCA) shown to be cytotoxic (15). Furthermore, recent studies have implicated bile acids in the pathophysiology of several neurological diseases, including Alzheimer’s, Parkinson’s, and Multiple Sclerosis (MS) (16).

### 2.2 Bile acid structure

It is important to note the differences between the human and mouse bile acid pool. While most of the human intestinal bile acid pool is composed of CA, CDCA, DCA and LCA along with their conjugated forms (17), the most highly abundant bile acids in the mouse pool are the primary bile acids α-muricholic acid (MCA) and β-MCA, which are generated from CDCA *via CYP2c70* (18). These differences in the bile acid pool should be considered when studying changes in bile acid synthesis and homeostasis. For example, the mouse pool of bile acids is more hydrophilic and less potent as detergents compared to the human pool, and depletion or expansion of certain bile acids may have differential effects (19). Steps have been taken to account for this challenge of making the mouse bile acid pool more like the human pool. For example, de Boer et al. generated a liver-specific *CYP2c70* knockout mouse model and found these mice to have a more hydrophobic bile acid pool similar to the human pool (20).

### 2.3 Bile acid transport

Both primary and secondary bile acids are actively reabsorbed in the distal ileum *via* the Apical Sodium-Dependent Bile Acid Transporter (ASBT) before circulating back to the liver (2). ASBT function is critical for maintaining the enterohepatic circulation of bile acids. In addition to ASBT, there are several other proteins in the intestine and liver that are crucial for proper bile acid transport. For example, following active transport *via* ASBT, bile acids bind to the intracellular bile acid binding protein (IBABP) and then exit the enterocytes by the organic solute transporters OSTα and OSTβ.

The hepatic secretion of bile acids is controlled by the ATP-binding cassette transporters bile salt export pump (BSEP) and multidrug resistance-associated protein (MRP2). While humans mainly use BSEP to efflux bile acids into the bile, mice use both BSEP and MRP2 (21). Bile acids are transported into hepatocytes mainly by the hepatic Sodium-taurocholate cotransporting polypeptide (NTCP). Additionally, the human organic anion-transporting polypeptides (OATP) OATP1B1 and OATP1B3 transport specific types of bile acids in the liver while excluding others. Specifically, CA, CDCA, and DCA are transported by these polypeptides, while the secondary bile acids LCA and UDCA are not (22). Furthermore, kinetic analyses revealed that conjugated bile acids are the preferred substrates as compared to unconjugated bile acids (23).

### 2.4 Bile acids and the gut microbiome

There are many dynamic interactions between gut bacteria and bile acids that depend largely on the expression of functional bacterial proteins, including bile acid transporters and modifying enzymes. For example, several proteins encoded by the bile acid inducible (*bai*) operon are involved in 7α -dehydroxylation of bile acids (24). Furthermore, the proton-dependent bile acid transporter (encoded by *baiG* gene) is responsible for the entry of primary bile acids into the bacterium, and the rate limiting step of 7α-dehydroxylation (25) is mediated by the bile acid 7α-dehydratase (*baiE*). Gut bacteria possess several other enzymes, such as the bacterial hydroxysteroid dehydrogenases (HSDs): α-HSD and β-HSD (26), which are responsible for oxidation and epimerization of bile acids at 3-, 7- and 12- positions in the steroid ring. The bacterial proteins known to interact with bile acids are widely distributed among different bacterial communities (27). Bile salt hydrolases, as well as oxidation and epimerization processes, have been identified in four different genera, including *Bacteroides, Clostridium, Bifidobacterium, and Listeria* (28).

There is an intricate and dynamic relationship between bile acid homeostasis and the gut microbiome that, when disrupted, can lead to several physiological complications. For example, the dietary supplementation of DCA in mice was shown to reduce the abundance of BSH-producing bacteria and increase the abundance of bacteria in the *Parabacteroides* and *Bacteroides* genera (29).

Recently, a novel class of human bile acids known as microbially conjugated bile acids (MCBAs) has been discovered (30). Unlike traditional conjugated bile acids, MCBAs are conjugated to the amino acids phenylalanine, leucine, and tyrosine (30). While the main species thought to produce these bile acids are *Enterocloster bolteae* and *Clostridium bolteae*, the underlying mechanisms behind the production of MCBAs have yet to be fully elucidated. The identification of these different bile acid species suggests that the gut microbiota plays a critical role in bile acid homeostasis and that alterations in bacterial composition significantly alter what species are present in the bile acid pool. Interestingly, the levels of MCBAs were shown to be elevated in patients with IBD and cystic fibrosis (30). However, the exact physiological roles of these newly discovered conjugated bile acids and how they contribute to diseases remain largely unknown.

## 3 Bile acids in IBD

Inflammatory bowel diseases (IBD) are multifactorial disorders of chronic intestinal inflammation consisting of two subtypes, Ulcerative Colitis (UC) and Crohn’s Disease (CD) (31). Ulcerative colitis is characterized by continuous lesions in the rectum and colon with inflammation localized to the mucosa and submucosa (32, 33). Crohn’s Disease, is characterized by skip lesions anywhere across the intestinal tract, most commonly affecting the terminal ileum, with full thickness inflammation (34, 35). Bile acids have been found to be altered in patients with IBD because of gut microbial dysbiosis (6). Generally, the observed trend is an increase in host derived bile acids, with a decrease in microbial derived secondary bile acids (6). Also, several lines of evidence support the notion that bile acid receptors modulate the immune response in IBD. The following sections review existing literature on the alterations in bile acids due to IBD as well as the directs effects of bile acids on IBD.

### 3.1 Bile acid receptors and immune responses

Bile acids are known to activate different receptors that were recently shown to affect immune responses as they relate to IBD (**Table 1**).

**Table 1:** Effects of Bile Acid Receptor Activation in Immune Cells.

Cell Type	Receptor	Effect	Ref
**Dendritic Cells (DCs)**	**FXR**	• ↓ IL-6, IL-1β, and TNF-α • ↓ migration to spleen in response to DSS colitis	(36–38)
	**TGR5**	• ↓ TNF-α and IL-12 production	(36, 37)
	**VDR**	• ↓ differentiation and maturation of DC	(36, 37)
**Natural Killer T-cells (NKTs)**	**FXR**	• ↓ IFN-γ and TNF-α, and ↑ IL-10 • Shift towards IL-10 producing NKT10 cells • ↓ production of osteopontin through SHP	(36, 37, 39, 40)
	**TGR5**	• ↓ IFN-γ and TNF-α • Shift towards IL-10 producing NKT10 cells	(36, 37, 39)
**Macrophages**	**FXR**	• ↓ IL-1β, TNF-α, NLPR3 inflammasome activity, and caspase 1 activity	(36, 37)
	**TGR5**	• Promotes differentiation from M1-like to M2-like • ↓ inflammasome activation	(36, 37, 41)
	**S1PR2**	• Polarization towards M1-like phenotype • ↑ IL-1β, NLPR3, pro-IL-1β, and pro-IL-18 • ↑ neutrophil migration and formation of neutrophil extracellular traps (NETs)	(42–46)
	**PXR**	• ↑ IL-1β and ATP release	(47)
	**VDR**	• ↓ IL-1, IL-6, IL-8, IL-12, and TNF-α • ↓ MLPR3 inflammasome, IL-1β secretion, and caspase 1 activation • ↑ ratio of M2:M1 macrophages	(36, 37, 48)
**T-Cells**	**VDR**	• ↓ T cell proliferation, • polarizes Th1 cells towards a Th2 phenotype • ↓ differentiation of Th17 cells	(48, 49)
	**RORγt**	• ↑ differentiation of Th17 cells • ↓ differentiation of Treg cells • ↓ IL-17 production	(3, 50, 51)
**Innate lymphoid cells (ILCs)**	**RORγt**	• ↑ differentiation and function of ILC3 subtypes	(3, 50)

↑ indicates an increase; ↓ indicates a decrease.

#### 3.1.1 FXR

FXR is expressed in cells of myeloid lineage including monocytes, macrophages, dendritic cells (DCs), and natural killer T-cells (NKTs) with different effects upon activation (52). FXR signaling in dendritic cells results in anti-inflammatory effects through decreases in IL-6, IL-1β, and TNF-α (36, 37). NKT cell-mediated activation of FXR also exhibits anti-inflammatory effects with decreases in interferon-gamma (IFN-γ) and TNF-α, and increases in IL-10 (36, 37). This signaling also shifts NKT cells towards an invariant NKT cell known as NKT10 cells, which produce IL-10 (39). These cells have been shown to decrease anti-tumor immune activity while also preventing autoimmunity in a mouse model of experimental autoimmune encephalomyelitis (EAE) (39). Furthermore, FXR activation in NKT cells has been shown to inhibit the production of osteopontin, a pro-inflammatory cytokine, through SHP (40). Generally, FXR utilizes small heterodimer partner (SHP) to bind to and sequester pro-inflammatory proteins (53). More specifically, SHP has been shown to inhibit the production of pro-inflammatory cytokines by decreasing NF-κB activation and mitogen activated protein kinase (MAPK) (54). FXR signaling in hepatic and intestinal macrophages exerts anti-inflammatory effects with decreases in the activation of IL-1β, TNF-α, NLPR3 inflammasomes, and caspase 1 (36, 37). One study utilized peripheral blood mononuclear cells (PBMCs) from healthy subjects, separated them into monocytes and dendritic cells, stimulated them with LPS, and treated them with INT-747, an FXR agonist, and found decreased TNF-α production (55). Overall, there is strong evidence that supports anti-inflammatory effects of FXR signaling.

These findings have been corroborated in various models of intestinal inflammation. A previous study utilized DSS and TNBS induced colitis in wild type and mice lacking FXR and showed that mice were treated with FXR agonist INT-747 exhibited decreased severity of colitis (55). This study also utilized lamina propria leukocytes from subjects with both ulcerative colitis and Crohn’s disease and found decreased secretion of IFN-γ, and TNF-α after treatment with INT-747. Follow up studies showed that obeticholic acid (OCA), a semi-synthetic bile acid used as medication and potent FXR agonist, prevented DC migration from the spleen to the colon in response to DSS-colitis with an increase in plasma IL-10 levels and IL-6 levels (38). It is interesting to note that IL-6 was also increased and that while less DC migration and increased IL-10 levels correspond with decreased inflammation, the increases in IL-6 demonstrate complex inflammatory responses resulting from FXR activation rather than binary pro- or anti-inflammatory effects. A retrospective study performed in a cohort of CD patients found that females carrying an FXR-1GT genotype had three times less FGF19 serum levels and had a higher risk of needing surgery, showing that impaired FXR signaling can be detrimental in IBD (56).

#### 3.1.2 GPBAR1/TGR5

G-protein bile acid receptor 1 (GPBAR1), also known as transmembrane G-protein-coupled receptor 5 (TGR5) is broadly expressed in various tissues and is also expressed in monocytes, macrophages, dendritic cells (DCs), and natural killer T-cells (NKTs) (57). TGR5 activation leads to decreases in pro-inflammatory IL-6, IFN-γ, and TNF-α, and increases in IL-10 (36, 37). TGR5 activation also promotes differentiation of macrophages from classically activated pro-inflammatory phenotypes to alternatively activated anti-inflammatory phenotypes (36, 37). In dendritic cells, TGR5 activation leads to decreased TNF-α and IL-12 production (36, 37). As with FXR, when TGR5 is activated, it promotes the polarization of NKT cells towards NKT10 cells, which produce the anti-inflammatory cytokine IL-10 (39). Additionally, activation of TGR5 in NKT cells also leads to decreases in IFN-γ, and TNF-α (36, 37). This body of evidence demonstrates important anti-inflammatory effects of TGR5 signaling in immune cells.

One recent study has shown that administration of TGR5 agonists during inflammatory stress protected against inflammasome activation. Treatment with Genistein, a compound known to protect against DSS colitis, increased intracellular cAMP levels in LPS treated macrophage cell lines (THP and U937 cells) (41). Furthermore, coadministration of the TGR5 agonist INT-777 with an NLRP3 inflammasome activator (Nigericin) increased the levels of intracellular cAMP and decreased secretion of caspase-1 and IL-1β, providing evidence for beneficial anti-inflammatory TGR5 signaling in macrophages (41). Another study showed that TGR5 activation by BAR501 ameliorated TNBS and oxazolone colitis by polarizing macrophages towards the less inflammatory alternative phenotype (58). The beneficial effect from BAR501 was not observed in TGR5 null mice (58). Further study using raw 264.7 cells showed that IL-10 activity was dependent on TGR5 (58). Another previous study showed that macrophage colony-stimulating factor (MCSF) and interferon-gamma differentiated macrophages challenged by enterococcus faecalis or LPS produced less TNF-α when treated with a TGR5 agonist (59). Furthermore, this study showed lamina propria macrophages from subjects with active Crohn’s disease had higher levels of TGR5 mRNA than those from healthy controls. The TGR5 agonist suppressed Enterococcus faecalis induced production of TNF α (59). Additional studies support a beneficial role for TGR5 signaling in the context of intestinal inflammation. TGR5 null mice showed increased susceptibility to severe colitis, and TNBS colitis mice treated with oleanolic acid, an TGR5 agonist, showed improvement (60). This evidence supports important roles for TGR5 signaling in inflammatory responses that should be further explored in additional clinically relevant models of inflammation. Collectively, it is interesting to note that activation of these two major bile acid receptors, FXR and TGR5, exerts anti-inflammatory response that could be exploited for therapeutic interventions for IBD.

#### 3.1.3 S1PR2

Bile acids are known to activate the sphingosine 1 phosphate receptor 2 (S1PR2). Many studies indeed, have shown the roles of S1PR2 in the modulation of immune responses. In this regard, Wang X et al. examined the effects of S1PR2 activation through LPS in primary murine macrophages (42). S1PR2 was found to be responsible for the increased protein expression of downstream targets RhoA and ROCK1. Administration of S1PR2 and ROCK1 antagonists was found to reduce the expression of CD86 and MCP-1 in an acute DSS model of colitis, suggesting that S1PR2 promotes polarization of macrophages to an M1/pro-inflammatory phenotype. This was followed up with *in vitro* studies where they used these antagonists in the presence of LPS and found that blocking S1PR2 and ROCK1 prevented increases in CD86, MCP-1, TNF-α, and iNOS mRNA expression in primary mouse macrophages providing further evidence that S1PR2 signaling in macrophages leads to a pro-inflammatory phenotype (42).

Additionally studies have shown that activation of S1PR2 results in a pro-inflammatory M1-like phenotype in human macrophages (61). Their data demonstrated treatment with an S1PR2 agonist led to an increase in the mRNA expression of pro-inflammatory M1 markers (nos2, cd80, cd86, il-1β, and il-12) in THP-1 derived macrophages challenged with Mycobacterium tuberculosis compared to those without S1PR2 activation. Furthermore, activation of S1PR2 in THP-1 derived macrophages during both Mycobacterium tuberculosis and Mycobacterium avium infections resulted in nitric oxide secretion as well as production of ROS. Regarding cytokine secretion, macrophages infected with Mycobacterium avium demonstrated increased IL-6 and decreased IL-10 secretion whereas those infected with Mycobacterium tuberculosis did not show differences in IL-6 secretion but did show increased secretion of TNF-α compared to untreated cells (61). Overall, this provides evidence that S1PR2 may play a role in response to infections. This provides an exciting opportunity to study the effects of bile acid mediated S1PR2 activation in the presence of infectious agents.

The roles of S1PR2 have also been explored in the context of murine cholestatic liver injury, where there is an increase in NLRP3 expression in bone marrow derived macrophages which translocated to the liver (43). Blocking S1PR2 with JTE-013 in bone marrow derived macrophages *in vitro* prevented an increase in the mRNA expression of NLRP3, pro-Il-1β, pro-IL-18, as well as IL-1β and IL-18 secretion. This was further supported by additional work by Hou L. et al (62) where it was found that activation of S1PR2 in murine bone marrow-derived macrophages led to the activation of the NLRP3 inflammasome. Specifically, S1PR2 activation with CYM-5520 led to secretion of IL-1β, and increased protein expression of NLPR3, pro-IL-1β, and pro-IL-18. This adds to the growing evidence that activation of S1PR2 in macrophages leads to a pro-inflammatory response.

There have also been extensive studies into the effects of S1PR2 signaling in neutrophils. Zhao X. et al. studied the roles of S1PR2 activation in neutrophils utilizing a neutrophil migration assay and found that inhibition of S1PR2 with JTE-013 in murine bone marrow neutrophils led to significantly less migration (44). S1PR2 antagonism also decreased neutrophil F-actin, suggesting that S1PR2 is important in neutrophil cytoskeletal remodeling. Similar results were found in the human dHL-60 cell line in which the same cell migration and cytoskeletal remodeling assays were performed. *In vivo* studies utilizing a mouse model of cholestatic liver injury showed that intraperitoneal (IP) administration of JTE-013, an S1PR2 antagonist, reduced neutrophil chemotaxis to the liver as measured through Lys6G mRNA expression and flow cytometry. Further work by Zhao X. et al. also found neutrophils can form neutrophil extracellular traps (NETs) in response to S1PR2 activation (45). The increased formation of NETs following S1P administration was confirmed *in vitro* through immunofluorescence of Cit-H3, neutrophilic elastase (NE), and myeloperoxidase (MPO). Furthermore, they applied S1P treatment in the presence of S1PR2 antagonist JTE-013 and found that the formation of NETs was blunted. *In vivo*, they administered S1PR2 siRNA in MCDHF (methionine and choline deficient high fat)-fed mice and found that blocking S1PR2 led to decreased levels of MPO-DNA complexes in the serum of these mice along with decreased protein expression of Cit-H3 in those livers, providing evidence for decreased neutrophil activation that was accompanied by decreased severity of liver injury. It was also found that S1PR2 was responsible for preventing spontaneous neutrophil apoptosis by administering S1PR2 antagonist JTE-013 and measuring the proportion of Annexin V positive cells (54). This finding suggests that S1PR2 activation in neutrophils also prolongs neutrophil life. Overall, this work adds to the growing body of evidence that S1PR2 is essential for pro-inflammatory neutrophil activation.

Another important aspect of S1PR2 signaling also involves intestinal epithelial cells as they possess important functions in immune regulation. Chen T. et al. examined the roles of S1PR2 signaling in intestinal epithelial cells utilizing intestinal epithelial cells (IECs) isolated from WT and S1PR2 KO mice both with and without acute DSS colitis (46). Co-culturing S1PR2^-/-^ IECs with CD4+ T-cells resulted in increased proliferation as compared to WT IECs. This suggests that in the presence of an inflammatory challenge, S1PR2 may be responsible for gatekeeping the amount of IEC mediated CD4+ T-cell proliferation. This was further supported with an increase in the mRNA expression of MHC-II in IECs from S1PR2 KO colitis mice compared to WT colitis mice suggesting that S1PR2 limits MHC-II expression and presentation to CD4+ T-cells. Interestingly, this study does not broadly agree with previous studies showing anti-inflammatory effects when S1PR2 was inhibited, however it suggests a more interesting phenomenon in which S1PR2 signaling exerts different effects depending on its cellular origin. In fact, two S1PR2 inhibitors, Etrasimod and Ozanimod have demonstrated potential as treatment options for individuals with ulcerative colitis, further highlighting the complexity of S1PR2 signaling in intestinal inflammation (63, 64).

#### 3.1.4 PXR

Pregnane X receptor (PXR) has also been shown to activated by bile acids and the roles of this receptor in altering immune responses was investigated. Hudson et al., studied the effect of PXR activation in LPS-primed macrophages by treating both murine peritoneal macrophages and THP-1 derived macrophages (47). Both mouse and human macrophages exhibited increased production of IL-1β after activation by species specific PXR ligands. Also, in both mouse and human macrophages lacking NLPR3, there was no induction of IL-1β production or activation of cacspase-1 after PXR activation, suggesting that PXR activation in macrophages is mediated through NLPR3. There were similar findings in human macrophages by showing that THP-1 derived macrophages treated with PXR agonists released ATP. While there is limited evidence compared to other bile acid receptors, this justifies further investigation into inflammatory responses after PXR activation.

#### 3.1.5 VDR

The Vitamin D Receptor (VDR) is not only highly expressed in the intestine but also in immune cells. Bile acids were shown to activate VDR, and this receptor is highly expressed in monocytes, macrophages, dendritic cells, and T cells. VDR activation in monocytes and macrophages generally results in anti-inflammatory effects with decreases in IL-1, IL-6, IL-8, IL-12, and TNF-α (36, 37). DC VDR activation results in the inhibition of the differentiation and maturation of dendritic cells. VDR activation in T-cells, results in the inhibition of T cell proliferation, and polarizes Th1 cells towards a Th2 phenotype (49). It also inhibits the differentiation of Th17 cells, which are known to play a role in the maintenance of the intestinal barrier in response to extracellular pathogens and increases the differentiation of tolerogenic Treg cells (3).

A recent study found that VDR activation by VitD3 suppressed MLPR3 inflammasome, IL-1β secretion, and caspase 1 activation in LPS challenged mouse peritoneal macrophages (48). This was confirmed *via* VDR siRNA silencing. Furthermore, VitD3 mediated VDR signaling reduced the severity of DSS colitis and found that it increased the ratio of M2 to M1 macrophages as well as inhibiting the differentiation of CD4+ T-cells into Th1 and Th17 T-helper cell subtypes. Wang et al. has also shown that VDR signaling protected from DSS colitis, providing additional evidence for VDR in immune signaling by shifting macrophages towards the M2 phenotype. He L., et al., utilized a TNBS model of colitis and found that mice lacking VDR in intestinal epithelial cells and specifically in colonic epithelial cells exhibited increased severity of colitis with increased Th1 and Th17 responses, and increased epithelial cell apoptosis with impaired gut permeability (65). Despite various studies demonstrating anti-inflammatory effects from VDR signaling, the direct influence of bile acids on VDR in the context of inflammation has not been clearly illustrated, providing an opportunity for important future studies.

#### 3.1.6 RORγt

Both Isoallo-LCA and 3-oxo-LCA are inverse agonists of RAR-related orphan receptor gamma (RORyt). In innate lymphoid cells (ILCs), RORyt activation leads to increased differentiation and function of ILC3 subtypes, which play an important role in mucosal homeostasis (3, 50). In T-cells, the activation of RORyt increases differentiation of Th17 cells and inhibits differentiation of Treg cells (51). In the case of the above mentioned oxo-bile acids, they bind RORyt as inverse agonists and in models of colitis, this results in decreased IL-17 and Th17 cells with a resulting improvement in inflammation.

A study with CD subjects showed that BI119, an RORyt antagonist inhibited the expression of Th17 genes in PBMCs challenged with bacterial antigens while simultaneously favoring expression of Treg genes (66). Furthermore, there was a decrease in pro-inflammatory mediators IL23R, CSF2, XCL1, CXCL8, and S100A8 and an increase in DEFA5, an antimicrobial protein, in ileal CD biopsy samples. RORyt inhibition in a T-cell transfer model of colitis also attenuated colitis. This offers evidence that RORyt has important roles in inflammatory responses, specifically in the intestines. While there is strong evidence that bile acids are playing key roles in RORγt signaling, the direct effects of specific bile acids on RORγt signaling remains understudied and thus remains an appealing area for future study.

### 3.2 Changes on bile acids and bile acid homeostasis in IBD

#### 3.2.1 Bile acid homeostasis

Several studies have extensively reported on alterations in bile acid signaling and bile acid levels in IBD. These studies are summarized in **Table 2**. Wilson A. et al., examined the changes in serum bile acid composition to study previously reported decreases in PXR and FXR activity in IBD patients (74). Both FXR and PXR reporter cell lines were treated with bile acid mixtures with the same composition found in human subjects (74). At a concentration of 75 μM, bile acid reporter activity was significantly decreased in the compositions corresponding to patients with CD for both FXR and PXR (74). 4βOHC was measured in their subjects as a marker of PXR mediated CYP3A4 activity and found them to be significantly lower in participants with CD (74). To look at FXR activity, FGF19 serum concentrations were measured and found to be significantly decreased in those with CD (74). Overall, this study provided strong evidence that the composition of the serum bile acid pool is significantly altered in Crohn’s Disease with a simultaneous alteration in bile acid homeostasis as evidenced by decreases in FXR and PXR signaling (74).

**Table 2:** Bile acid alterations in IBD.

Bile Acid	Compartment	Model/Population	Changes	Ref
CA	Serum	CD with ileocecal resection CD Pediatric UC	• ↑ • ↓ postprandial increase • ↑ postprandial levels	(67–69)
	Feces	UC UC & CD Identical twins with ileal CD	• ↑ • ↑ • ↑ 3α, 7α, and 12α-trihydroxy-5β -cholanate	(27, 70–72)
	Colon Fluid	UC	• Non-significant ↓	(73)
CDCA	Serum	CD Pediatric UC	• ↑ in ileocecal resection CD vs CD and control • ↔ • ↓ postprandial increase • ↑ postprandial levels in active UC	(67, 68, 74)
	Feces	UC IBD Pediatric IBD	• Non-significant ↑ • ↓ • ↑ • ↑	(70, 71, 75)
DCA	Serum	CD	• ↔	(74)
	Feces	UC CD	• ↓ • Non-significant ↓ • ↓ in unoperated ileal CD with further decrease after surgery	(71, 76)
	Bile	CD	• ↓	(77–81)
LCA	Serum	UC CD	• ↓ • ↓	(67, 74)
	Feces	CD IBD	• ↓ in unoperated ileal CD with further decrease after surgery • Non-significant ↓ • ↓, ↓ 12-ketoLCA	(71, 75, 76, 82)
UDCA	Serum	UC	• ↑ in UC subjects with hepatobiliary symptoms compared to UC subjects without	(67)
	Bile	CD	• ↓ • Non-significant ↑ • ↑ • ↑ in ileal resection compared to healthy controls	(79–81, 83)
TCA	Serum	UC CD	• ↑ in UC subjects with hepatobiliary symptoms compared to UC subjects without • ↓ total tauroconjugated • ↓	(67, 74)
	Feces	UC CD	• ↑ • ↑	(71, 72)
TCDCA	Serum	CD	• ↔	(74)
	Feces	UC	• ↓	(71)
TLCA	Serum	CD	• ↔	(74)
	Feces	UC CD	• ↓ • ↓	(71, 75)
TUDCA	Serum	UC	• ↑ in UC subjects with hepatobiliary symptoms compared to UC subjects without	(67)
GCA	Serum	UC CD	• ↑ in UC subjects with hepatobiliary symptoms compared to UC subjects without • ↑ in proportion • ↓ total glycoconjugates • ↓ post-prandial increase	(67, 68, 74)
	Feces	UC	• Non-significant ↑	(71)
GCDCA	Serum	UC CD	• ↑ in UC subjects with hepatobiliary symptoms compared to UC subjects without • ↓ proportion	(67, 74)
	Feces	UC CD	• ↑ • ↑	(71)
GDCA	Serum	CD	• ↑ in proportion	(74)
	Feces	UC	• ↓	(71)
GLCA	Feces	UC	• ↓	(71)
HDCA	Serum	IBD	• ↑	(67)

↑ indicates an increase; ↓ indicates a decrease; ↔ indicates no change.

Another key aspect of bile acid homeostasis is the diversity of the bile aicd pool which is heavily influenced by bacterial transformation. Das P. et al. performed shotgun metagenomics to study bile salt transformation genes on the phyla level in the gut microbiota of healthy subjects and those with IBD (27). Subsequently, they investigated how these changes corresponded to levels of fecal bile acids. The abundance of bile salt biotransformation genes (BSBGs) was significantly lower in subjects with CD compared to healthy subjects. This was further examined by mapping each gene to its corresponding taxonomic lineage and found that the majority of BSBGs corresponded to the phylum Firmicutes with consistent decreases in the abundance of Firmicutes in subjects with IBD.

Another study examined bile acid metabolism directly related to the gut microbiota by applying pure bile acid solutions to stool homogenates followed by direct measurement of chemically modified bile acids. The microbiota of healthy subjects was able to perform deconjugation, transformation, and desulphation on bile acids, whereas those capabilities were diminished in the IBD samples. These findings demonstrate how IBD induced gut microbial dysbiosis directly affects bile acid composition (84).Rutgeerts P. et al. examined colonic CD subjects and found that the bile acid pool size was significantly decreased in CD subjects when compared to healthy subjects and to subjects with UC (77). Another study from the same group found that CD subjects with ileal involvement maintained a normal level of fasting total bile acids, whereas those with additional colonic involvement did not, suggesting that the colon played a role in the maintenance of the total bile acid pool and that this process was disrupted in CD with colonic involvement (85). Koga T. et al., showed that in patients with CD, the administration of diet higher in fat led to greater excretion of fecal bile acids, providing evidence for bile acid malabsorption caused by CD (86). Rutgeerts P. et al., showed that subjects with CD experiencing bile acid malabsorption experienced an increase in duodenal cholesterol saturated bile (78).

Another important area of study has been measuring changes in metabolism of bile acids in IBD. Tougaard L. et al., examined the turnover rate of orally administered [14]-C labeled TC by measuring fecal excretion of CA, CDCA, DCA, and LCA in order to study bile acid metabolism in CD subjects with terminal ileal involvement (87). There was increased turnover of CA and DCA in CD subjects, along with decreases in CA and DCA in unoperated subjects. Those who had undergone ileal resection showed normal levels and presumably underwent compensatory increases in the synthesis of CA and DCA.

Furthermore, most patients with IBD are on medical treatment, a variable that is often confounding, but Roda G. et al., specifically looked at serum bile acid levels in IBD subjects who were treated with anti-TNF agents (88). Based on a bile acid profile of up to 15 different bile acids, various patient populations can be distinguished. PCA analysis showed separation between anti-TNF treated and untreated CD subjects, UC subjects, and healthy controls. CD patients on anti-TNF treatments showed an increase in total serum bile acids compared to CD patients that did not receive those treatments. This was driven by an increase in secondary bile acids. Ultimately, anti-TNF agents used to treat IBD may re-establish physiological levels of bile acids that are disturbed during IBD.

There are additional variables which need to be considered in the study of bile acid homeostasis disruptions in IBD such as age. Jacobs J.P. et al., recruited families with pediatric IBD cases to study gut microbial structure and associated metabolic changes (89). The bile acids CA, 7-ketoDCA, sulfated CDCA, and 3-sulfoDCA were associated with CD. Martin F.P., et al. studied urinary bile acid profiles in pediatric subjects with IBD and found that subjects with IBD excreted less urinary GHDCA (90). IBD subjects also excreted higher levels of urinary THCA, UDCA, TUDCA, and B -UCA. In general, there are few studies on pediatric cohorts with IBD and additional rigorous studies are warranted to determine if there are age specific alterations in bile acid homeostasis. Collectively, changes in bile acids in IBD patients are mainly mediated by the associated dysbiosis.

#### 3.2.2 Conjugated and unconjugated bile acids

As previously mentioned, a key aspect in bile acid homeostasis involves the conjugation of bile acids and several changes in conjugation have been reported in IBD. Gnewuch C. et al. found decreases in total serum unconjugated bile acid levels in UC (67). Those with CD also exhibited a higher ratio of serum conjugated to unconjugated bile acids when compared to healthy subjects (74). Duboc, et al., found increases in conjugated bile acids in those with IBD, and an increase in sulphated forms of bile acids only in those with active disease when compared to healthy controls (84). Vantrappen G et al. found a decrease in the total size of the bile acid pool with an increased ratio of glycine conjugated bile acids to taurine conjugated bile acids in CD subjects when compared to healthy controls (83). The reduction in bile acids was related to the activity of the disease. Linnet K., et al. studied the effects of CD on fasting and postprandial serum bile acids (91). After a meal, the concentrations of taurine conjugated bile acids were lower in CD than in healthy controls. On the other hand, there was no difference in glycine conjugated bile acids. Additionally, the ratio of total glycine to taurine in serum bile acids was increased in CD subjects than in healthy controls in both fasting and postprandial states. Tanida N. et al. also found that there was an increase in the fecal excretion of total bile acids in both wet and dry fecal compartments in UC subjects (92). Additionally, glycine and taurine conjugated bile acids were higher in diseased subjects compared to healthy controls. Further, unconjugated and sulfated bile acids were decreased in UC feces compared to healthy controls. These changes were exacerbated in those with increased disease severity. Ejderhamn J. et al. examined fecal bile acid excretion in a pediatric population of IBD (70). Bile acids in the feces and in fecal water were measured and demonstrated that subjects with IBD had increased levels of total bile acids, unconjugated bile acids, as well as glycine and taurine conjugated bile acids in their feces. In the fecal water, total bile acid excretion was increased, including increases in taurine conjugated bile acids with concentrations 2 to 5-fold higher in IBD subjects. In subjects with clinical remission of their disease, increased taurine conjugated bile acids in both the feces and fecal water compartments were observed. Overall, these findings suggest a decrease in bacterial Bile Salt Hydrolase (BSH) activity in IBD patients leading to a decline in the relative abundance of unconjugated bile acids.

##### 3.2.2.1 Serum

Several studies have measured the levels of unconjugated bile acids in the serum of subjects with IBD under various states (**Table 2**). One study observed that in CD subjects who had undergone ileocecal resection, serum CA and CDCA were increased when compared to controls and CD subjects that had not undergone surgery (67). Heuman R. et al. measured fasting and postprandial serum levels of CA and CDCA conjugates in IBD (68). The average postprandial increase in CA and CDCA was significantly decreased in subjects with CD compared to healthy subjects. Additionally, subjects who had undergone surgical resection for their disease exhibited a more subdued postprandial increase in CA with no differences in CDCA. These studies suggest that there is a lack of absorption of bile acids due to ileal resection and damage from CD. Furthermore, this may lead to a compensatory increase in the hepatic production of CA and CDCA.

In order to explore why patients with IBD typically have less PXR and FXR activity, Wilson A. et al., examined their changes in serum bile acid composition (74). They found no differences in serum proportion of CDCA and decreased proportions of LCA between healthy controls and subjects with inactive or active CD. They also found no differences in serum DCA levels between healthy subjects and those with CD (74). Gnewuch C. et al. (2009) found a decrease in serum LCA in both a CD and UC cohort (67). LCA and UDCA were increased in UC subjects with hepatobiliary issues compared to those without. This is a very interesting finding that warrants further mechanistic inquiry. Overall, a decrease in LCA levels in subjects with IBD falls in line with our understanding that microbes are needed to transform primary bile acids and those microbes can be lost during IBD.

In a study of pediatric IBD, Ejderhamm J. et al. found that subjects with active UC exhibited significantly higher levels of CA and CDCA in their serum, four hours after food consumption when compared to healthy, age-matched subjects (69). These findings suggest either increased absorption of primary bile acids in the small intestine perhaps as a compensatory mechanism for impaired colon function or a decrease in the bacterial transformation of these bile acids due to microbial dysbiosis, however this is purely speculative and there may be additional physiologic variables due to this being a pediatric cohort.

Overall, these studies demonstrate how changes in bile acid during IBD are context specific. While conducting such studies would be challenging, further clinical investigations with larger sample sizes and granular stratifications based upon age, severity of disease, surgical history, and intestinal region affected, are warranted to allow for better understanding of how unconjugated bile acids specifically are being altered in both UC and CD.

##### 3.2.2.2 Feces

While there are a few studies looking at serum levels of unconjugated bile acids in IBD, there are several more doing so in the feces. Yang, Z.H., et al. found an increase in fecal CA with decreases in DCA, LCA, and 12-ketoLCA, in UC subjects compared to healthy controls, while also reporting trends towards increases in CDCA (71). Additionally, DCA, LCA, 6-ketoLCA, and 12-ketoLCA were negatively correlated with serum levels of IL-1α, IL-1β, TNF-α, and IL-6. These findings support mainstream understanding of bile acids in IBD where the abundance of primary bile acids is increasing either due to increased hepatic production, decreased intestinal absorption, and or a concurrent decrease in their microbial transformation to secondary bile acids. Further, increases in secondary bile acids during lower levels of pro-inflammatory cytokines also support less microbial dysbiosis during less inflammation.

An understudied area in the measurement of fecal bile acids involves colon fluid. Vertzoni M. et al. collected colon fluid endoscopically in UC subjects with UC in relapse and in remissions and analyzed the composition of the fluid (73). There were no significant differences in bile acids between subjects in relapse compared to those in remission. The authors surmised that the lack of apparent difference may have been due to low statistical power. Additionally, CA trended towards a decrease in subjects with a relapse in their UC compared to those in remission, although this was not significant. While these findings are unremarkable, there is less precedence for the use of colon fluid to measure bile acids in IBD subjects and models of IBD. Nevertheless, these findings are important to note and perhaps they will be augmented by future studies which may justify the use of colon fluid for bile acid measurements in IBD.

Jansson et al., performed non-targeted metabolic profiling in identical twin pairs including healthy subjects and subjects with CD and in the case of ileal CD, found increases in fecal 3α, 7α, and 12α-trihydroxy-5β -cholanate (72). Fiasse R. et al. analyzed fecal bile acid levels in subjects with ileal CD before and after ileal resection (82). DCA and LCA were decreased in unoperated ileal CD as compared to healthy controls, with a further reduction after surgery. The authors speculated that the decrease in DCA corresponded to decreased transit time in CD subjects and thus having a decreased exposure to bacterial 7α-dehydroxylase. It is also possible that the loss of microbes possessing 7α-dehydroxylase activity may contribute to these observations.

Studies by Das P. et al. found that subjects with IBD had reduced levels of BSBGs and proceeded to examine fecal bile acid compositions in these subjects and found that CA was increased while LCA was decreased in IBD subjects (encompassing both UC and CD) (27). Ejderhamn J. et al. examined fecal bile acid excretion in a pediatric population of IBD (70). In the fecal water, CA and CDCA were increased with concentrations 2 to 5-fold higher in IBD subjects. Weng Y.J., et al. examined the interactions between diet, microbiota, and metabolites in patients with IBD and found that CDCA and LCA were significantly decreased in the feces of patients with both UC and CD (75). The species Fusobacterium nucleatum was also associated with CDCA and LCA levels in patients with CD. Franzosa E., et al., performed untargeted metabolomic and shotgun profiling in subjects with IBD and healthy controls (76). They found significant increases in CDCA in the stools of IBD subjects. They also found non-significant decreases in DCA and LCA in CD subjects. It is challenging to draw strong conclusions for the changes in fecal levels of unconjugated bile acids from the studies surveyed. This is due to several factors including small sample sizes which force the grouping of UC and CD subjects together even though both subsets of IBD have distinct pathophysiology. As with the studies in serum, rigorous studies with larger sample sizes across the various manifestations in IBD are needed. However, these will most likely require cross-disciplinary and multi-institutional collaborations with strong clinical implications for their findings to justify the studies in the first place.

##### 3.2.2.3 Bile

A less investigated compartment for the measurement of bile acids in IBD is in the bile itself, due to the practical challenges of more invasive techniques needed for samples. Due to procedural challenges, these studies are not as common and are less current, but they offer an important insight, nevertheless. Many of these studies were performed by Dr. Rutgeerts and most were performed in subjects with CD.

In one study by Rutgeerts, studying cholic acid metabolism it was found that the metabolism of CA and CDCA was significantly increased in subjects with ileal CD when measured directly from the bile, and to those with both ileal and colonic involvement when compared to healthy subjects (85).

Nishida, T. et al., orally administered deuterated CDCA in subjects with CD and healthy volunteers (79). Using duodenal bile samples, they measured a significant decrease in the biological half-life of CDCA, the pool size of CDCA, and the total bile acid pool in the bile of subjects with CD. They also found a non-significant decrease in DCA and a non-significant increase in UDCA levels in CD when compared to healthy subjects.

Rutgeerts P. et al. examined duodenal bile in subjects with CD localized to the colon and found significantly decreased concentrations of DCA in subjects with CD compared to healthy controls (77). Another study by them found that in subjects with CD experiencing bile acid malabsorption, bile acid composition in bile had significant decreases in DCA when compared to healthy subjects (78).

Lapidus A. et al. found that bile acid composition in the duodenal bile of subjects with CD exhibited a significant decrease in the abundance of DCA and UDCA (80). In another study they found that in CD subjects with ileal resection, there was a significant decrease in DCA, but significant increase in UDCA levels in duodenal bile compared to healthy controls (81). Vantrappen G., et al., found that CD subjects had significant reductions of UDCA in their bile (83).

These studies highlight an exciting avenue for further investigation in pre-clinical models where the invasiveness of sampling bile does not pose the same concerns it does in humans even if it poses additional technical challenges. Few physiological studies have explored the changes in bile acid composition specifically in the bile in models of IBD. Such studies could provide important mechanistic insight into these clinical observations that have already been made.

#### 3.2.3 Taurine conjugated bile acids

##### 3.2.3.1 Serum

Overall, there are few studies examining the levels of taurine conjugated bile acids in the serum of IBD cohorts. Gnewuch C. et al. (2009) found a decrease in serum total bile acid tauroconjugates in a CD cohort, along with a general decrease in total bile acids (67). They also found an increase in serum TCA and TUDCA in UC subjects with hepatobiliary symptoms compared to UC subjects without hepatobiliary issues. Another study found that patients with CD also had significant decreases in the proportion of serum TCA, but no changes in TLCA (74). This limited evidence suggests that patients with CD have an impaired conjugation ability.

##### 3.2.3.2 Feces

There are also few studies looking at fecal levels of taurine conjugated bile acids in IBD. Yang, Z.H., et al. found an increase in fecal TCA, but a decrease in TCDCA and TLCA in UC subjects compared to healthy controls (71). Furthermore, TCA was positively correlated with serum IL-α and TNF-α., while TLCA was negatively correlated with serum levels of IL-1α, IL-1β, TNF-α, and IL-6. Jansson et al., performed non-targeted metabolic profiling in identical twin pairs including healthy subjects and subjects with CD and in the case of ileal CD, found increases in fecal TCA (72). Weng Y.J., et al. found that TLCA was significantly decreased in the feces of patients with both UC and CD (75). While this is not extensive evidence, these findings suggest that fecal TCA may be associated with disease activity and its microbial transformation is diminished during IBD most likely due to gut microbial dysbiosis.

#### 3.2.4 Glycine conjugated bile acids

##### 3.2.4.1 Serum

There are also limited studies on serum glycine conjugated bile acids in IBD. Gnewuch C. et al. (2009) found a decrease in serum total bile acid glycoconjugates in their CD cohort (67). In UC subjects with hepatobiliary symptoms, GCA and GCDCA was increased compared to UC subjects without hepatobiliary issues. Wilson A. et al., examined the changes in serum bile acid composition to study observed decreased in PXR and FXR activity in IBD patients, and found significant decreases in the proportion of GCDCA with significant increases in the proportion of GCA and GDCA in the in the serum of subjects with both inactive and active CD as compared to healthy controls (74). Suchy F.S., et al. looked at postprandial serum bile acid concentrations in subjects with ileal CD (68). There were greater increases in GCA and total bile acids after a meal in healthy subjects when compared to those with CD. These studies are important to note, but given their differences in context, it is challenging to reach a unified conclusion.

##### 3.2.4.2 Feces

In the feces, Yang, Z.H., et al. found an increase and decrease in GCDCA and GLCA respectively in UC subjects compared to healthy controls (71). Furthermore, GCDCA was positively correlated with serum IL-α levels, while GDCA was negatively correlated with serum levels of IL-1α, IL-1β, TNF-α, and IL-6. They also found a trend towards an increase in fecal GCA in UC subjects compared to healthy controls, although it did not reach statistical significance (71). Also, GCA was positively correlated with serum IL-α. Jansson et al., performed non-targeted metabolic profiling in identical twin pairs including healthy subjects and subjects with CD and found that subjects with CD had higher levels of fecal GCDCA (72). These findings both demonstrate an increase in primary glycine conjugated bile acids in the feces of IBD subjects. This may be due to either decreased absorption and/or increased hepatic synthesis.

### 3.2.5 Primary and secondary bile acids

Another key variable to consider when examining alterations in bile acids during IBD is the difference in abundance between primary and secondary bile acids because of the gut microbial dysbiosis that is known to occur in IBD. Yang, Z.H., studied the gut microbial profiles in patients with UC and fecal bile acid levels (27). Briefly, 32 UC subjects and 23 age and sex matched healthy controls were recruited and stark differences in their microbiota was found. PCA analysis showed a clear separation between those subjects with UC and healthy controls. Additionally, measures for α diversity such as the Chao1, Shannon, and Simpson diversity indexes showed decreased diversity in those with UC. Further analysis demonstrated significant alterations in both phylum, genus, and species compositions. There was a significant decrease in the amount of overall secondary bile acids in the feces of patients with UC. Immunohistochemistry on mucosal biopsy samples demonstrated a significant increase in the expression of bile acid receptor TGR5 and a significant decrease in VDR in UC subjects. UC subject serum also had increased levels of IL-1α, IL-1β, TNF-α, IL-2, and IL-6. Overall, this study found that fecal bile acid profiles were strongly associated with gut microbial and serum cytokine profiles, demonstrating that bile acids are strongly impacted by the systemic inflammation and microbial gut dysbiosis that occurs in IBD and UC more specifically.

Kruis W. et al. found that the daily fecal bile acid excretion rate was increased in subjects with CD and decreased in UC compared to healthy controls (93). CD and UC subjects had significant increases in primary bile acids but decreased amounts of secondary bile acids as compared to healthy controls. At the time, the authors speculated that the decrease may have been attributed to faster gastrointestinal transit time in UC subjects and increased acidity in CD feces. However, given recent findings, it seems possible to speculate that these changes may also occur due to microbial dysbiosis.

Duboc, et al., studied the effects of IBD induced gut microbial dysbiosis on bile acid metabolism by measuring the levels of bile acids in both feces and serum in healthy subjects, active IBD, and IBD in remission (84). There was a decrease in secondary bile acids abundance in subjects with IBD in both serum and feces. Das P. et al. found that subjects with IBD had reduced levels of BSBGs and proceeded to examine fecal bile acid compositions in these subjects and found that the primary bile acid CA was increased in IBD subjects (encompassing both UC and CD), and LCA was low (27). Levels of conjugated bile acids GCA, TC, and TCDA were elevated in IBD subjects compared to healthy controls. They also found decreases in secondary bile acids in IBD subjects when compared to healthy controls.

## 4 Effects on bile acids on intestinal epithelial cells and intestinal inflammation

Most studies of bile acids and intestinal epithelial cells utilized cell lines which lack other cell types found in the intestines while also lacking some physiological aspects that are present in newer primary enteroid cell culture systems and thus the findings of such studies need to be carefully interpreted. Overall, studies examining the effects of bile acids on intestinal epithelial cells would be greatly improved with the utilization of more complex models such as primary intestinal cultures and primary intestinal cultures co-cultured with immune cells to help delineate cell specific mechanisms that are often obscured *in vivo* but not captured with less complex *in vitro* models. There have been several studies examining the effects of individual unconjugated bile acids on intestinal epithelial cell TEER, apoptotic signaling, and cytokine secretion.

### 4.1 Unconjugated bile acids

One such study by Duboc H. et al., studied the effects of unconjugated bile acids on IL-1β induced IL-8 secretion in a Caco-2 cell culture model (84). While CA and CDCA did not alter IL-8 secretion, DCA and LCA inhibited the IL-1β mediated secretion of IL-8. LCA had a dose-response relationship with IL-1β mediated IL-8 secretion and treatment with sulfated LCA negated the anti-inflammatory effect. Another study utilized murine colonic epithelial cells to mimic wounds found in ulcerative colitis in an *in vitro* scratch model. Ultimately, CA, CDCA and LCA had no effect intestinal epithelial cell growth in response to the *in vitro* wound in the form of a scratch (94). However, DCA impaired epithelial cell growth in response to the insult. Sarathy et al. looked at the effects of bile acids on the production of inflammatory cytokine IL-8 in T84 cells. They found a five-fold increase in IL-8 production from CDCA, and an inflammatory cocktail led to a 21-fold increase (95). On the other hand, LCA led to a 50% reduction in IL-8 production in response to the inflammatory cocktail.

### 4.2 Conjugated bile acids

**Figure 1:**
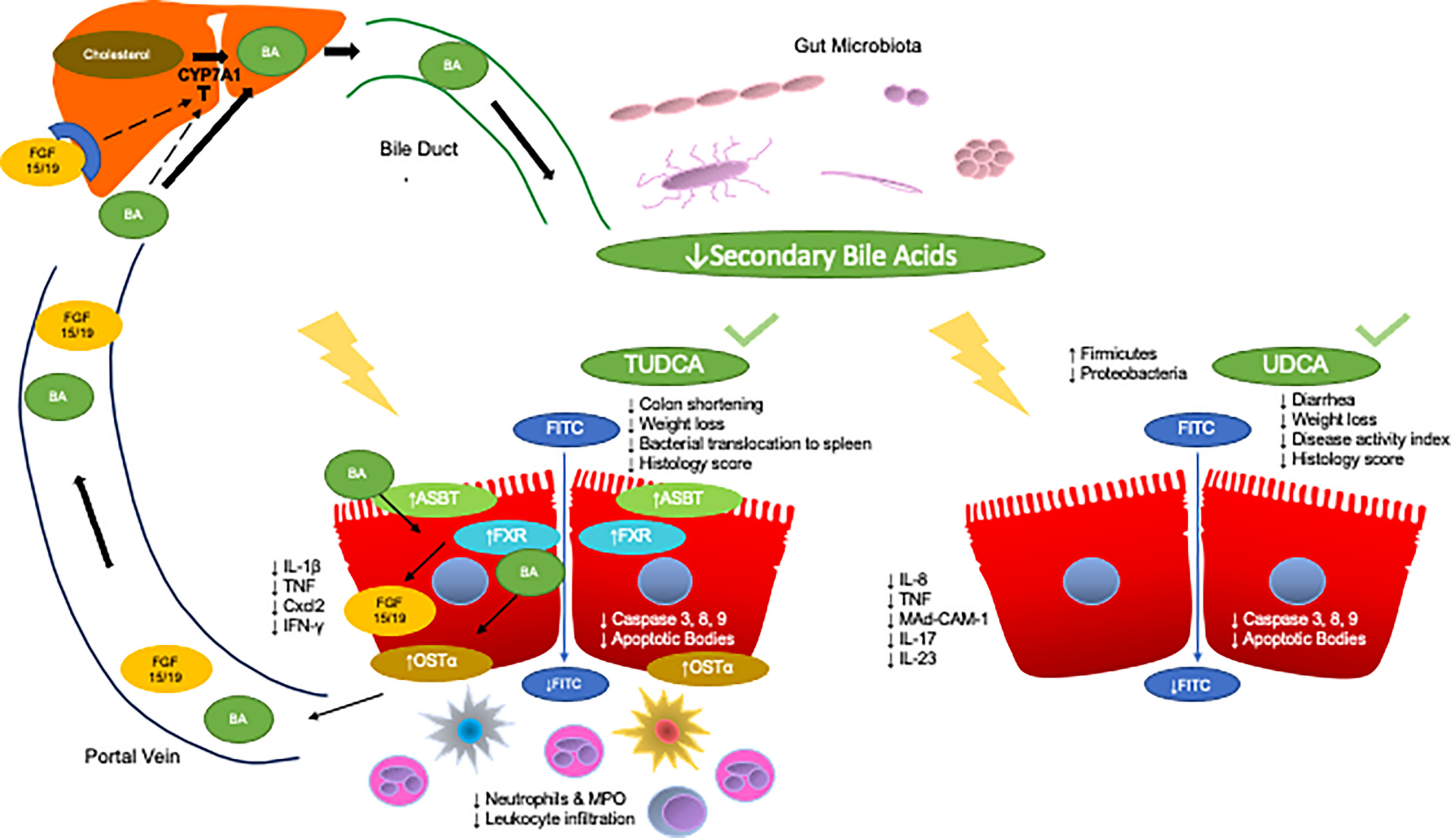
Anti-inflammatory effects of TUDCA and UDCA in models of IBD. The enterohepatic circulation of bile acids transports bile acids synthesized and modified in the liver to the intestines through the bile duct. The rate limiting step of hepatic synthesis of bile acids is mediated by the CYP7A1 enzyme which is inhibited by bile acids and ileal FGF15/19. Gut microbes then convert primary bile acids into secondary bile acids. Gut microbial dysbiosis in IBD leads to a reduction in the secondary bile acid pool. Specifically, two key secondary bile acids have been shown to exert anti-inflammatory effects in IBD and relevant models, TUDCA and UDCA. Administration of TUDCA on cell culture and mouse models of IBD aided in bile acid homeostasis by preventing loss of OSTα expression as well as increased FXR and ASBT expression. Furthermore, TUDCA protected against colon shortening, weight loss, and led to improvements in histological score. TUDCA also led to the reduction of pro-inflammatory cytokines IL-1β, TNF, Cxcl2, and IFN-γ as well as decreased apoptotic signaling. Furthermore, TUDCA led to decrease neutrophilic infiltration and activity, as well as general decreases in leukocyte infiltration. Administration of UDCA led to less diarrhea and weight loss as well as decreases in disease activity index and histology score. UDCA also led to decreased apoptotic signaling. Furthermore, UDCA led to decreases in IL-8, TNF-α, and Mad-CAM-1 in the colon, and less IL-17 and IL-23 in the serum of subjects with UC as well as increases in the abundance of Firmicutes and decreases in Proteobacteria.

TUDCA, a secondary bile acid, has been shown to have anti-inflammatory potential (**Figure 1**). Van den Bossche L. et al., studied the effects of TUDCA on TNF-α treated Caco-2 cells and TNF-α overexpressing mice CD with ileal involvement (96). TNF-α treated Caco-2 cells demonstrated decreased mRNA expression of PXR and OSTβ with trends towards a decrease in the expression of FXR, VDR, ASBT, and OSTα. TNF-α also led to increased IL-8 secretion and decreased TEER. Administration of TUDCA to apical compartments prevented TNF-α induced reduction of PXR and OSTα expression and trended towards an increase in expression of FXR and ASBT. VDR and OSTβα did not change in response to TUDCA. Surprisingly, TUDCA also resulted in a synergistic decrease in TEER.

The effects of TUDCA on intestinal epithelial cells have also been explored in additional cell lines. HT29 and T84 cells lines were challenged with TNF and co-administered TUCDA (97). TUDCA prevented a significant reduction in the induction of caspases 3, 8, and 9 in HT29 cells. In T84 cells, they found that TUDCA prevented the formation of apoptotic bodies as assessed by electron microscopy. They also found that co-administration of TUDCA with FasL in HT29 cells prevented FasL induction of caspase-3 activation. To find the pathway responsible for these findings, the role of the α5β1 integrin receptor (which can sense TUDCA in hepatocytes) was studied and found that blocking that peptide did not interfere with TUDCA protection against caspase-3 activation, keeping this mechanism unknown.

## 5 Bile acids as therapeutic agents for intestinal inflammation

### 5.1 Preclinical models

There is limited data on bile acids as therapeutics in human IBD, but several studies have been performed in preclinical animal models. These data as well as human studies are summarized in **Table 3**. In a model of indomethacin induced enteropathy, Shibuya N. et al. found that DCA exacerbated small intestinal inflammation (98). DCA increased expression of ICAM-1 and VCAM-1 which facilitated the adhesion and thus the migration of lymphocytes to the ileum. This effect was due to S1PR2 signaling as an S1PR2 antagonist diminished DCA induced increases in ICAM-1 and VCAM-1 expression *in vitro* and diminished the indomethacin and DCA induced intestinal damage.

**Table 3:** Effects of bile acids on inflammatory responses.

Bile Acid	Model	Effects	Ref
Cholic Acid (CA)	• IL-1β treated Caco-2 cells	• ↔	(84, 94)
	• Mouse colonic epithelial cell scratch assay	• ↔	
Chenodeoxycholic Acid (CDCA)	• T84 Cells	• ↑ IL-8 •	(84, 94, 95)
	• IL-1β treated Caco-2 cells	• ↔	
	• Mouse colonic epithelial cell scratch assay	• ↔	
Deoxycholic Acid (DCA)	• IL-1β treated Caco-2 cells	• ↓ IL-8	(84, 94, 98–100)
	• Mouse colonic epithelial cell scratch assay	• ↓ cell growth	
	• Indomethacin induced small intestinal inflammation	• ↑ ICAM-1 and VCAM-2 • ↑ lymphocyte migration	
	• Acute DSS Colitis (Mouse)	• ↑ NLPR3 inflammasome and S1PR2 signaling	
Lithocholic Acid (LCA)	• T84 Cells	• ↓ IL-8 • ↑ barrier function • ↓ apoptotic signaling	(84, 94, 95, 101)
	• IL-1β treated Caco-2 cells	• ↓ IL-8	
	• Mouse colonic epithelial cell scratch assay	• ↔	
	• Acute DSS Colitis (Mouse)	• ↑ barrier function • ↓ apoptotic signaling • ↓ histology score	
Ursodeoxycholic Acid (UDCA)	• TNBS Colitis (Rat)	• ↓ alkaline phosphatase • ↓ affected area • ↑ colon IL-1β	(101–106)
	• DSS treated MDR2^(-/-)^ model of cholestatic liver injury and colitis	• ↓ severity of colitis • ↓ MAdCAM-2	
	• DSS Colitis	• ↑ barrier function • ↓ apoptotic signaling • ↓ histology score • ↓ weight loss • ↓ diarrhea • ↓ disease activity index	
	• T84 cells	• ↓ TNF-α release • ↑ intestinal barrier integrity • ↓apoptotic signaling	
	• Case report of Crohn’s Disease patient	• Development of enterolith	
	• Ulcerative Colitis subjects on Mesalazine	• ↓ Mayo score • ↓ Mayo endoscopic score • ↑ quality of life scores • ↓serum IL-23 and IL-17 • ↑ gut Firmicutes • ↓ gut Proteobacteria	
Taurocholic Acid (TCA)	• TNBS colitis (Mouse)	• ↑ mortality • ↓ weight loss • ↓ colon shortening • ↓ histological score • ↓ colon MPO • ↓ TNF-α, IL-1β, and IFN-γ	(107)
Tauroursodeoxycholic Acid (TUDCA)	• TNF-α treated Caco-2 cells	• Prevented ↓ OSTα • ↑ FXR and ASBT • Synergistic ↓ TEER	(96, 97, 107–109)
	• Acute DSS colitis with recovery, Tollip ^(-/-)^ Neutrophils (Mouse)	• ↓ colon shortening • ↓ weight loss • ↓ activated neutrophils	
	• Acute DSS colitis with two-week recovery (Mouse)	• ↑ mortality • ↓ IL-1β, Cxcl2, TNF • ↓ bacterial translocation to spleen • ↑ anti-apoptotic signaling • ↓ decreased apoptotic signaling • ↓ LPS barrier dysfunction	
	• TNF treated HT29 cells	• ↓ induction of caspases 3, -8, and -9	
	• TNF treated T84 cells	• ↓ apoptotic bodies	
	• TNBS Colitis (Mouse)	• ↓ weight loss • ↑ mortality • ↓ histological score • ↓ TNF-α, IL-1β, IFN-γ, and MPO • ↑ colon length, • ↓ Grp78	
	• TNF^ARE^ mice	• ↑ FXR, ASBT, OST-α • ↑ weight • ↓ histological score • ↓ leukocyte infiltration scores	
Taurohyodeoxycholic Acid (THDCA)	• TNBS Colitis (Mouse)	• ↓ weight loss • ↓ histological damage • ↓ COX-2, MPO activity • ↓ serum TNF-α and IL-6	(110)

↑ indicates an increase; ↓ indicates a decrease; ↔ indicates no change.

Studies by Ward J. et. al., showed that intraperitoneal administration of LCA and UDCA were protective against acute DSS colitis (101). Disease activity was assessed by measuring intestinal epithelial permeability through FITC-dextran uptake and by measuring apoptosis through capsapse-3 cleavage. LCA improved intestinal barrier function and reduced apoptotic signaling. Furthermore, histological scores were reduced by both LCA and UDCA However, additional parameters used to measure the severity of colitis such as weight loss and colon shortening were not significantly altered by LCA or UDCA. Similar findings were observed after LCA treatment during inflammation in a T84 colonic epithelial cell culture model treated with TNF-α and IFN-gamma. They found improvement in epithelial barrier integrity and apoptosis signaling in response to LCA and UDCA during inflammation. An interesting aspect of their studies was the use of a chemical analogue of UDCA which is not capable of being metabolized, 6-methyl-UDCA, and found this compound had no effect in any of their studies.

The role of secondary bile acids, specifically UDCA has also been explored in chemically induced models for IBD (**Figure 1**). Martinez-Moya P., et al., studied the effects of UDCA on a TNBS induced colitis rat model (102). Administration of UDCA at a dose of 50 mg/kg day through an esophageal catheter resulted in protective effects whereas there were no significant effects at lower doses. Rats treated with UDCA exhibited decreased alkaline phosphatase activity as well as a smaller affected area. However, UDCA treatment did lead to an increase in colonic IL-1β production compared to control rats where this was not observed with TNBS treated rats.

In a co-model for cholestatic liver injury and colitis in the form of multidrug resistance protein 2 (MDR2) knockout mice treated with DSS, it was found that the bile acid UDCA reduced the severity of colitis and downregulated colitis susceptibility protein mucosal addressin-cell adhesion molecule 1(MAdCAM-1) (103). Further, previous studies have also established UDCA and its precursor, LCA, protected against DSS colitis *in vivo* by preventing weight loss, restoring fecal pellet formation, and attenuating disease activity index (104). Similar effects we observed *in vitro* in cytokine treated colonic epithelial cells as measured by decreased TNF-α release.

The role of bile acid conjugation has also been studied specifically in the cases of taurine conjugated bile acids. Taurocholic acid (TCA), a conjugated primary bile acid has been shown to exert anti-inflammatory effects in chemically induced colitis. Yang Y., showed that oral gavage of TCA prevented TNBS weight loss and improved mortality (107). TCA also prevented colon shortening and increased colon weight due to TNBS colitis. Benefits were also observed on a histological level, where mice treated with TCA had a lower histological score on average than those without. TCA treatment during TNBS colitis also resulted in decreased colonic MPO, TNF-α, IL-1β, and INF-gamma levels. More recently, Wong, W.Y. et al. showed that increasing the amount of taurine conjugated bile acids through Lactobacillus case strain Shirota (LcS) mediated increases led to improvement in DSS induced colitis (111). Specifically, improvement in colon length, improvement in histological architecture, and decreases in CD45 expression in the colons of these mice as compared to untreated mice with DSS colitis. LcS treatment also improved expression of Occludin on the protein level, and Muc2 on the mRNA level. This treatment and this improvement coincided with serum increases in TCA and TCDCA in LcS treated mice compared to untreated DSS colitis mice. There were also serum decreases in α- and β-MCA after LcS treatment. While this was not a direct treatment with bile acids, this study provides evidence for beneficial effects from taurine conjugated bile acids. This was evident as TCA and TCDCA level increases were accompanied by decreases in IFN-gamma and increases in IL-10 mRNA in the colons of DSS colitis mice treated with LcS as compared to untreated colitis mice.

While there is evidence that taurine conjugated bile acids are protective against DSS colitis, there is additional evidence in other models for intestinal inflammation. TNF-α overexpressing mice given TUDCA in their water for eleven weeks showed benefits in their bile acid homeostasis (96). TUDCA treatment increased the mRNA expression of FXR with a trend towards an increase in VDR, and no change in PXR. TUDCA also resulted in increased mRNA expression of bile acid transporter genes ASBT, and OST-α, with no change in OST-β. Furthermore, they performed principal component analysis (PCA) utilizing the expression data from their selected bile acid homeostasis genes and found that TUDCA treated TNF^ARE^ mice shifted closer towards wild type mice than TNF^ARE^ mice who did not receive TUDCA. TUDCA treated mice demonstrated continued weight gain through 13 weeks of age as compared to TNF^ARE^ mice which began to exhibit weight loss at 11 weeks of age. The histological score and leukocyte infiltration scores were also significantly decreased in TNF^ARE^ mice treated with TUDCA. This study provides strong evidence that TUDCA provides some anti-inflammatory effects in a mouse model for Crohn’s ileal inflammation while also restoring some aspects of bile acid homeostasis that are impaired in ileitis.

In a model of acute DSS colitis (7-day DSS treatment followed by 7-day recovery period) with Tollip (Toll-interacting protein) deficient neutrophils, Diao N., et al. showed that DSS colitis was more severe in mice with neutrophils lacking Tollip (toll-interacting protein) (108). They examined the roles of TUDCA on DSS colitis in WT mice because it is known to restore Tollip function. Mice treated with intraperitoneal TUDCA did not exhibit colon shortening or experience as much weight loss. TUDCA also reduced the amount of circulating activated neutrophils as measured through the cell surface expression of C14. This finding is in line with previous studies by Laukens D. et al., where they also demonstrated that TUDCA inhibited the effects DSS colitis (97). In this study, all mice treated with intraperitoneal TUDCA survived for a full two weeks after DSS administration. Mice treated with TUDCA exhibited a significant reduction in weight loss and a reduction in colon shortening 10 days post-DSS. Furthermore, there was significantly less IL-1β protein in the colon 10 and 14 days post-DSS. There was also significantly less TNF expression on post-DSS day 10 as well as significantly reduced Cxcl2 expression after 7, 10, and 14 days post-DSS. TUDCA protected against intestinal epithelial barrier dysfunction by measuring less bacterial translocation to the spleen. Furthermore, they found that in colonocytes isolated from mice treated TUCDA, there was a significant decrease in Caspase-3 activity on day 3 and 14 post-DSS. There was also an increase in the expression of Bcl2 on days 3 and 10 post-DSS. Additionally, pre-treatment with TUDCA before challenge with LPS resulted in less intestinal barrier dysfunction as measured through FITC-dextran and less enterocyte apoptosis in the ileum.

The effects of TUDCA have also been studied in other models of colitis. Yang Y., et al., studied the effects of TUDCA on TNBS colitis and found that it protected against colitis during a seven-day treatment beginning 24 hours after rectal TNBS administration (23). Administration of TUDCA through oral gavage prevented TNBS colitis weight loss and improved survival rate. TUDCA also significantly improved histological score and resulted in a significant decrease in colon levels of TNF-α, IL-1β, IFN-gamma, and MPO. This study adds to the available evidence that TUDCA is protective against chemically induced colitis.

More recently, Long Y., et al., showed that treatment of TNBS colitis mice with TUDCA demonstrated improved colon length, and decreased histological score (109). To explore potential reasons for these beneficial effects from TUDCA, Grp87, an endoplasmic reticulum stress marker that they found was increased in patients with Crohn’s Disease, was studied. Treatment with TUDCA prevented Grp78 upregulation in response to colitis through immunohistochemistry. This suggests TUDCA exerts its positive effects by relieving ER stress. The performed subsequent studies *in vitro* to show that ER stress can promote an inflammatory response through the activation of the p38 MAPK pathway, but they did not directly test the effects of TUDCA during those experiments.

Taurohyodeoxycholic acid (THDCA) was investigated by He J. et al. in a model of TNBS colitis (110). They used THDCA extracted from Pulvis Fellis Suis and administered it through oral gavage for seven days. THDCA alleviated weight loss due to colitis, improved macroscopic score, and improved histological architecture. There was a decrease in COX-2 expression assessed by immunohistochemistry in the colons of mice treated with THDCA. Furthermore, there were decreases in MPO activity in the colon, as well as decreased circulating TNF-α and IL-6 levels in the serum. Studies in other models of IBD may further elucidate the evidenced anti-inflammatory properties of THDCA.

### 5.2 Human subjects

While there have been extensive studies on bile acids as potential therapeutics for IBD in preclinical models, there are limited studies examining these effects in human IBD. Wang, Z. et al., has examined the roles of UDCA in conjunction with Mesalazine on patients with ulcerative colitis. In this prospective, single center study, participants with UC were randomly assigned to two arms: treatment with Mesalazine or treatment with Mesalazine and UDCA (105). Subjects in the two groups had comparable baseline Mayo scores, but one-week post treatment, those receiving UDCA had significantly lower Mayo scores. With regards to endoscopic Mayo score, those on Mesalazine alone did not show any changes even four weeks post treatment. However, those also receiving UDCA had significantly lower Mayo endoscopic scores four weeks post treatment compared to their baseline and compared to the first week post treatment. Subjects were also administered an IBD questionnaire to assess their quality of life. Four weeks post treatment, scores of social ability, emotional ability, systemic ability, and overall total score were increased in those receiving UDCA and Mesalazine compared to those receiving Mesalazine alone. Furthermore, serum IL-23 and IL-17 levels were also lower four weeks post treatment in those receiving UDCA and Mesalazine compared to those taking Mesalazine alone. Additional microbiota profiling revealed that those who received UDCA in addition to Mesalazine had higher abundance of Firmicutes and lower abundance of Proteobacteria. While this study offers promising evidence of UDCA exerting beneficial effects in human UC, this study showed limited information on the severity of the disease and most importantly was only conducted for four weeks, and thus longer-term data is vital.

While there is evidence for benefits in UDCA as an adjunct treatment for IBD, those benefits may come with unintended consequences. Matsui H., et al. reported on a case of a Crohn’s disease patient who developed an enterolith after UDCA administration (106). This patient had achieved remission of her Crohn’s disease with prednisolone, azathioprine, and infliximab, and maintained it with azathioprine and infliximab. After her diagnosis with Crohn’s disease, the patient had been diagnosed with hepatic dysfunction for which she received oral UDCA. Ultimately, the enterolith was removed surgically with jejunal resection and it was found that 98% of the enterolith was made up of UDCA. While purely speculative, they suspect that the UDCA precipitated and in combination with intestinal stasis in the jejunum resulted in the formation of this enterolith. While this is a case study, this study is important to note.

Overall, care must be taken in analyzing these studies due to the nuances between the various models. *In vitro* studies modeling intestinal epithelial cells have shown that CDCA increased IL-8 secretion while LCA diminished IL-8 in two distinct human intestinal epithelial cell lines. However, to the best of our knowledge, CDCA alone was not explored in animal models and thus conclusions about CDCA remain limited. On the other hand, LCA appeared to improve intestinal barrier function and reduce apoptotic signaling in a chemical colitis model which coincides well with *in vitro* data. This was also the case with UDCA where there was strong evidence for anti-inflammatory properties when considering studies performed *in vitro*, in preclinical animal models, and in humans (**Figure 1**). Conversely, DCA showed a reduction in *in vitro* IL-8, but promoted lymphocyte migration and inflammatory signaling in mouse models of intestinal inflammation, highlighting the need for identification of cell specific sources of inflammation in such studies.

Furthermore, there is evidence that suggest anti-inflammatory effects of taurine conjugated bile acids (**Figure 1**). In several studies and various models including pro-inflammatory challenged intestinal epithelial cell lines, chemical induced colitis, and genetic models of enteropathy, there were overall decreases in the production of pro-inflammatory cytokines, weight loss and histological damage. These studies suggest a beneficial role for taurine conjugated bile acids in the context of intestinal inflammation that should be further explored as a therapeutic option.

## 6 Bile acids and barrier function/intestinal permeability

IBD are multifactorial disorders characterized by genetic susceptibility, immune cell overactivation, microbial gut dysbiosis, and changes in intestinal barrier permeability (112). The following section summarizes available literature on the studies of bile acids directly modulating intestinal barrier function *in vitro*, in animal models for IBD, and in human and animal intestinal explant studies using a Ussing chamber (**Table 4**). Overall, there is substantial evidence that for bile acids are modulating intestinal permeability through inflammatory changes, and alterations in tight junctions.

**Table 4:** Effects of bile acids on intestinal permeability.

Bile Acid	Model	Effect	Ref
CA	Caco-2	• ↔ TEER • ↔ TEER (apical); ↓ TEER and ↑ mannitol flux (basal) • ↓TEER, ↑ mannitol flux	(113–115)
	Porcine jejunal explants	• ↑ permeability (lucifer yellow) • disruption of cellular architecture (EM)	(116)
CDCA	T84	• ↓ TEER, ↑ permeability (10 kDA Cascade Blue dextran) • ↓ Occludin and disrupted localization	(95)
	Caco-2	• ↓ TEER and ↑ IL-8 • ↓ TEER and ↑ mannitol flux (apical and basal) • ↓ TEER and ↑ fluorescent dextran flux, disrupted Occludin architecture	(113–115)
	IPE-J2	• ↓ TEER and ↑ ZO-1, Occludin, and claudin-1	(117)
	Human sigmoid colon biopsies	• ↓ TEER and ↑ uptake of chemically killed E. coli	(118)
DCA	Caco-2	• ↓ TEER and ↑ permeability of phenol red • ↓ TEER • ↓ TEER and ↑ flux of fluorescent dextran • ↓ TEER; ↑ IL-1β, IL-6, TNF-α; ↓ ZO-1	(15, 113–115, 119, 120)
	Human sigmoid colon biopsies	• ↓ TEER and ↑ permeability (CR-EDTA)	(118)
	Mouse intestinal tissue	• ↑ FITC dextran flux, disruption of Occludin architecture	(121)
LCA	T84	• ↔ TEER; attenuated ↓ TEER from CDCA and cytokines	(95)
	Caco-2	• ↑ TEER after LPS • Prevented ↓ in TEER and ↑ in FITC-dextran permeability from TNF-α	(120, 122)
UDCA	Caco-2	• ↔ TEER • ↔ TEER or paracellular flux	(113, 115)
	Indomethacin induced intestinal inflammation (Rat)	• Protected against ↑ FITX dextran permeability	(123)
	Mouse intestinal tissue	• ↔ FITC dextran permeability; protected against DCA induced barrier dysfunction	(121)
	Mouse experimental peritonitis	• ↓ LPS induced FITC dextran flux	(124)
TCA	Caco-2	• ↓ TEER and ↑ mannitol flux	(114)
	Rat	• ↑ ileal permeability	(125)
TDCA	Caco-2	• ↓ TEER and ↑ mannitol flux	(114)
TUDCA	IPE-J2	• Reversed E. coli induced ↓ in ZO-1, claudin-1, and Occludin	(126)
	Weaned Piglets	• ↑ Occludin, claudin-1 in jejunum • ↓ diarrhea • ↓ circulating LPS and DAO	(126)

↑ indicates an increase; ↓ indicates a decrease; ↔ indicates no change.

### 6.1 Unconjugated bile acids

Horikawa T., et al., looked at the effects of bile acids on intestinal barrier function in differentiated Caco-2 cells (113). While CA and UDCA had no effect and DCA induced a slight decrease in TEER, CDCA lead to a greater than 50% reduction in TEER with a concomitant increase in IL-8 secretion. This was further studied with time and dose-dependent studies and found decreases in TEER, increase in FITC-dextran measured permeability, and IL-8 secretion at their highest concentration of CDCA treatment. While the greatest reduction in TEER happened at 8 hours, the greatest increase in IL-8 secretion and mRNA expression occurred after 24 hours. To investigate mechanistically, they performed similar treatments in the presence of FXR inhibitor guggulsterone. While guggulsterone did not reverse CDCA mediated decreases in TEER, it did block CDCA induced release of IL-8, IL-6, and TNF-α, but not VEGF. FXR inhibition additionally prevented CDCA induced mRNA expression of IL-8 and IL-6. This work provides evidence that CDCA directly modulates intestinal barrier function at least partially through FXR signaling.

Lowes S. et al. studied the effects of cholic acid on human intestinal epithelial Caco-2 and T84 cells (114). Apical administration of cholic acid on Caco-2 monolayers did not affect TEER over time. However, basal administration of cholic acid led to a quick decrease in TEER and demonstrated dose-dependent responses on TEER. This alteration was further characterized in intestinal barrier function by measuring mannitol flux and found that basal cholic acid increased mannitol flux through the Caco-2 monolayers. Furthermore, basal administration of DCA led to a quick decrease in TEER and demonstrated dose-dependent responses on TEER. They further characterized this alteration in intestinal barrier function by measuring mannitol flux and found that basal DCA increased mannitol flux through the Caco-2 monolayers. They also found that basal administration of CDCA reduced TEER and increased mannitol flux in both Caco-2 and T84 cells.

Raimondi F. et al. challenged human intestinal epithelial Caco-2 cells with various bile acids and found that CA, CDCA, and DCA administration led to a significant temporary decrease in TEER (115). Additionally, CA facilitated a transient increase in the paracellular flux of fluorescent dextran, while CDCA also resulted in a loss of organized structure in the tight junction protein Occludin. UDCA had no effects.

Danielsen E.M. et al. studied the effects of sodium cholate on the intestinal barrier in porcine jejunal explants (116). Administration of sodium cholate led to an increase in permeability as assessed by lucifer yellow fluorescence. However, only some enterocytes were demonstrating this increase in permeability and that the brush border marker Aminopeptidase N (ApN) stayed localized to the apical surface and the Na+/K+-ATPase stayed localized to the basolateral membrane suggesting that there were no disturbances in the membranes in response to sodium cholate. This was further assessed by electron microscopy and found that while sodium cholate did not affect the columnar shape of enterocytes or the length of the microvilli in the brush border, there was a disruption of the dense hexagonal structures observed in untreated explants.

Munch et al. studied the effects of DCA on intestinal permeability in human sigmoid colon biopsies with Ussing chambers and found that DCA and CDCA led to significant decreases in TEER (118). Furthermore, CR-EDTA was utilized as a marker of paracellular permeability and found that DCA demonstrated a dose-dependent response on CR-EDTA permeability. DCA and CDCA also increased the uptake of chemically killed E. coli.

Sarathy J. et al. studied the effects of CDCA in human intestinal epithelial T84 cells and found that CDCA decreased TEER (95). Furthermore, they studied the effects of CDCA with a pro-inflammatory cytokine cocktail consisting of TNFα, IL-1β, and IFNγ. The inflammatory treatment led to a reduction in TEER, with a further reduction in combination with CDCA. Apical administration of CDCA increased paracellular permeability and in conjunction with the pro-inflammatory cocktail it led to a greater increase in paracellular permeability measured with 10kDA Cascade Blue dextran. LCA did not cause any changes in paracellular permeability alone, but it did attenuate the increases due to CDCA. Additionally, in combination with both CDCA and the pro-inflammatory cocktail, LCA also attenuated the increase in paracellular permeability observed during administration of the pro-inflammatory cocktail and CDCA. CDCA did not alter claudin-2 protein expression, but it decreased Occludin expression. Specifically, immunohistochemistry showed a disruption of Occludin localization patterns. LCA did not alter claudin-2 protein expression and treatment with LCA did not prevent the CDCA induced disruption on Occludin. Similar protective effects for LCA were observed by others. In this regard, Yao B. et al. examined the effects of LCA on intestinal barrier function in a human epithelial Caco-2 model challenged by TNF-α (122). Pre-treatment with LCA prevented TNF-α induced increases in FITC-Dextran permeability as well as TNF-α induced decreases in TEER. LCA prevented TNF-α mediated decreases in the protein levels of ZO-1, E-cadherin, Occludin, and Claudin-1. Furthermore, they studied the localization of these tight junction proteins with immunofluorescence and found that LCA attenuated TNF-α mediated disruptions in tight junction protein localization.

Song M. et al. examined the effects of CDCA on LPS induced intestinal epithelial barrier function (117). CDCA treatment reversed LPS induced increases in TEER as well as LPS induced decreases in tight junction proteins ZO-1, Occludin, and Claudin-1 in IPEC-J2 porcine intestinal epithelial cells. In mice, CDCA administration prevented LPS mediated increases in intestinal permeability as measured by serum FITC-dextran as well as LPS induced decreases in tight junction proteins ZO-1, Occludin, and Claudin-1.

Stenman L.K. et al. investigated the effects of DCA and UDCA on mouse intestinal tissues in Ussing chambers (121). While UDCA had no effect, DCA exhibited a dose dependent response on the impairment of barrier function in both the jejunum and the colon as assessed by 4kDa FITC dextran flux. DCA also increased permeability to larger 20kDA FITC dextran in the colon and not in the jejunum. Although, co-administration of UDCA with DCA protected against DCA induced barrier dysfunction in the colon but not in the jejunum. DCA also altered cellular distribution of Occludin assessed through immunofluorescent imaging without altering the protein amount assessed through Western blot. Co-administration of LPS with DCA exacerbated DCA induced barrier function in the colon of both mice and rats while LPS alone had no effect on the flux of FITC dextran. *In vivo*, a diet supplemented with 0.1% DCA also impaired gut barrier function as assessed by serum FITC dextran.

Golden Jamie M. et al. studied the effects of UDCA on the intestinal barrier in a model of murine experimental peritonitis induced by intraperitoneal LPS (124). They found that UDCA significantly decreased LPS induced increases in gut permeability through a FITC-dextran assay. Additionally, they found that LPS treatment led to a shortening of villus height that was partially prevented with UDCA.

Liu L. et al. studied the effects of DCA in Caco-2 and IMCE (Immorto-Min colonic epithelium) cells (119). DCA led to decreases in TEER in Caco-2 cells. DCA also increased expression of pro-inflammatory cytokines IL-1β, IL-6, and TNF-α in both Caco-2 cells and IMCE cells. In mice, DCA led to a decrease in the expression of tight junction protein ZO-1 on an mRNA levels and through immunofluorescence.

Zeng H. et al. (2022) found similar findings with DCA on Caco-2 cells (15). DCA led to increases in permeability of phenol red as well as a decrease in TEER. On a protein level, DCA led to a dose dependent decrease in Occludin expression. Furthermore, Occludin decreases were found from DCA treatment with immunofluorescence as well. Bile acids have also been shown to directly alter intestinal barrier function in the context of inflammatory bowel disease (IBD). One study utilizing a co-culture of Caco-2 and HT29-MTX-E12 cells with LPS activated dendritic cells (DCs) in the basolateral compartment showed that activated DCs impaired barrier function through decreased TEER (120). Subsequently, LCA and sulfated DCA administration can marginally restore TEER. While there are several studies demonstrating how bile acids directly alter intestinal barrier function, a key factor in IBD, additional studies are warranted to better understand which bile acids are beneficial, which are not, and why they are leading to their respective alterations in intestinal permeability.

Bernardes-Silva et al. studied the effects of UDCA on intestinal permeability in a rat model of indomethacin induced intestinal inflammation (123). Intestinal permeability was assessed by measuring urine levels of chromium-51-ethylenediaminetetraacetate (42) (CR-EDTA) after oral gavage. Administration of UDCA in conjunction with the offending agent indomethacin prevented indomethacin induced increases in intestinal permeability to the point where no differences were found between rats who received UDCA and indomethacin versus the controls.

With the exception of LCA, most of the available literature points to unconjugated bile acids as being disruptors or having no effect on intestinal barrier function in both *in vitro* studies with models of intestinal epithelial cells and explants, and *in vivo* models of intestinal inflammation.

### 6.2 Taurine conjugated bile acids

Lowes S. et al. studied the effects of TCA and TDCA on human intestinal epithelial Caco-2 and T84 cells (114). Basal administration of TCA led to a quick decrease in TEER and demonstrated dose-dependent responses on TEER. This alteration in intestinal barrier function was assessed by measuring mannitol flux and found that basal TCA increased mannitol flux through the Caco-2 monolayers. Basal administration of TDCA also reduced TEER and increased mannitol flux. Furthermore, there were similar effects on T84 cells with T84 cells being more sensitive to bile acid mediated disruption of their barrier integrity.

Liu H. et al. studied the effects of CA supplementation on intestinal barrier integrity in rats (125). There was an accumulation of TCA at the ileum and subsequently found that TCA at concentrations found in CA fed mice increased ileal permeability in a Ussing chamber but not in other regions of the intestine.

There have also been important studies that have occurred in porcine models. Song M., et al. examined the effects of TUDCA specifically on the intestinal barrier function of weaned piglets (126). They administered TUDCA for 30 days to newly weaned piglets and found reduced incidence of diarrhea. Histological examination of the jejunum and ileum showed increases in the villus height to crypt depth ratio and in the proportion of PAS positive goblet cells per villus with TUDCA supplementation. There were increases in the expression of tight junction proteins Occludin and Claudin-1 but not ZO-1 at a protein level in the jejunum. Further, there were decreased levels of circulating LPS and diamine oxidase (DAO) in the serum. This evidence supports the finding that TUDCA directly influences intestinal permeability. Additionally, TUDCA exerted a beneficial effect on systemic and intestinal immunity. TUDCA reduced IL-1β and NF-kB while increasing IL-4 and IL-10 in the ileal mucosa. This was accompanied by an increase in serum IgG and secreted IgA in ileal mucosa. This provides direct evidence that TUDCA is directly modulating intestinal immunity in a porcine model.

Song M., et al. also examined the effects of TUDCA *in vitro* in porcine intestinal columnal epithelial IPEC-J2 cells challenged with *K88* E*. coli* to induce epithelial barrier dysfunction (126). Treatment with TUDCA reversed the *E. coli* induced decreased in ZO-1, Claudin-1, and Occludin protein expression while also reducing LDH activity in the cell culture supernatant. Additionally, TUDCA treatment with and without *E. coli* led to significant increases in the expression of bile acid receptor TGR5, but not in FXR. Subsequently, the beneficial TUDCA effects were mediated through TGR5 signaling by knocking down TGR5 and showing that without TGR5, TUDCA did not improve *E. coli* disruption of epithelial barrier function *in vitro*. This was also supported by their data showing that during the TGR5 knockdown, TUDCA did not lead to increased levels of cAMP. This was further explored by looking at the MLCK pathway. Intestinal epithelial cells challenged with *E. coli* had increased MLCK expression along with an increase in the ratio of phosphorylated to unphosphorylated MLC. TUDCA treatment prevented this MLCK pathway activation, but when TGR5 was knocked down, this did not occur further providing evidence that the beneficial effects of TUDCA on intestinal permeability are mediated by TGR5. This was further supported with a MLCK inhibitor and observed similar effects compared to TUDCA.

There is an interesting juxtaposition between the effects of taurine conjugated bile acids on intestinal barrier function and their effects on IBD or models of IBD. Most taurine conjugated bile acids showed beneficial effects towards intestinal inflammation. However, when relating to barrier function, the data suggests that taurine conjugated bile acids mostly impair intestinal barrier function. This is counterintuitive as intestinal permeability is thought of as a hallmark of IBD and as a marker of disease severity especially in pre-clinical models. These contradictions warrant further investigation and possibly even suggest that perhaps the field needs to rethink the way that intestinal permeability is linked to disease activity in IBD.

## 7 Conclusion

The roles of bile acids in modulating the immune responses are emerging. There is collective evidence from multiple studies and experimental approaches to support their disturbances in cases of intestinal inflammation. Unique alterations in the composition of bile acid pool are attributed to dysbiosis associated with IBD. Yet, the mechanisms by which bile acids contribute to the pathophysiology and/or the severity of intestinal inflammation are not fully understood. The complex nature of bile acid homeostasis and presence of several receptors expressed in different cell types that could be activated by bile acids make it challenging to delineate the causal link between bile acids and the inflammatory response in IBD. Future research in this field should leverage advanced bioinformatic and systems biology approaches to gain novel insights about their potential roles in the pathophysiology of IBD. Also, the profound differences in the composition of bile acid pool between humans and rodents raise serious concerns about the extent to which data obtained from these models could be extrapolated to humans. The generation of transgenic mouse models with bile acid pool similar to that of humans should offer excellent preclinical models to facilitate new discoveries related to bile acids and IBD. Further, better understanding of how gut microbiota shapes the response to a changing pool of bile acids remains a major interest of investigations. Since data suggest that a single bacterial modification of bile acids may rather involve communities of different bacteria from distinct species, future studies should focus on investigating the changes in specific microbial consortia in IBD and their effects on bile acid homeostasis. Additionally, investigating the novel roles of the newly discovered microbially conjugated bile acids will likely yield to novel findings that could further explain the potential roles of gut microbiota/bile acids axis in the development of intestinal inflammation. Overall, the increase in our knowledge about bile acids as inflammatory mediators will enhance our understanding of IBD and will define novel targets for improved therapeutic modalities for treatment of these debilitating gut disorders.

## Author contributions

NC and SC performed initial literature screening. NC, SC, RKG, and WAA finalized the first draft. NC created tables and Figure. NC, SC, PKD, SS, RKG and WAA edited and approved final version. All authors contributed to the article and approved the submitted version.
